# *Pseudomonas aeruginosa* bacteremia in patients with liver cirrhosis: a comparison with bacteremia caused by Enterobacteriaceae

**DOI:** 10.1186/1471-2334-13-332

**Published:** 2013-07-19

**Authors:** Ji Hwan Bang, Younghee Jung, Shinhye Cheon, Chung Jong Kim, Kyung Ho Song, Pyeong Gyun Choe, Wan Beom Park, Eu Suk Kim, Sang Won Park, Hong Bin Kim, Myoung-don Oh, Hyo-Suk Lee, Nam Joong Kim

**Affiliations:** 1 Department of Internal Medicine, Seoul National University College of Medicine, 101 Daehak-ro Jongno-gu, Seoul, 110-744, Republic of Korea; 2 Division of Infectious Diseases, Seoul Metropolitan Government - Seoul National University Boramae Medical Center, Seoul, Republic of Korea

**Keywords:** *Pseudomonas aeruginosa*, Bacteremia, Liver cirrhosis

## Abstract

**Background:**

This study was performed to detect risk factors for *Pseudomonas aeruginosa* bacteremia in patients with liver cirrhosis.

**Methods:**

A retrospective case–control study was designed to identify risk factors for *P*. *aeruginosa* bacteremia in cirrhotic patients. The cases were cirrhotic patients with *P*. *aeruginosa* bacteremia and the controls were cirrhotic patients with Enterobacteriaceae bacteremia.

**Results:**

Sixty-one cases and the same number of controls were enrolled. In a multivariate analysis, younger age {adjusted odds ratio (aOR) per one year: 0.96, 95% confidence interval: 0.93 - 0.99}, nosocomial acquisition (aOR 3.87, 95% confidence interval: 1.50 - 9.94), preexisting biliary disease (aOR 4.79, 95% confidence interval: 1.92 - 10.47), and recent exposure to immunosuppressive agent (aOR 3.10, 95% confidence interval: 1.23 - 7.82) were associated with *P*. *aeruginosa* bacteremia. In the case group the frequency of appropriate initial antibiotic regimens was considerably lower than in the control group: 29.5% vs. 65.6% (*P* <0.01). However, thirty day mortality did not differ significantly between cases and controls (19.7% vs. 24.6%).

**Conclusions:**

Nosocomial acquisition, preexisting biliary disease, and recent use of immunosuppressive agents are strong predictive factors for *P*. *aeruginosa* bacteremia in cirrhotic patients.

## Background

Blood stream infection by Gram negative bacteria is a common complication in cirrhotic patients, and the prognosis of the bacteremia in this group is poor [1,2]. Third generation cephalosporins are most commonly used to treat infectious complications in patients with liver cirrhosis, but their antimicrobial spectra against *Pseudomonas aeruginosa* are not identical to each other [3-5]. It has been reported that inadequate or delayed antibiotic therapy for pseudomonas bacteremia is associated with poor prognosis [6-9]. Hence, empirical antimicrobial therapy that lacks anti-pseudomonal activity could be harmful in case of pseudomonas bacteremia.

Thus, it is important for clinicians to be aware of the risk factors for pseudomonas bacteremia. Neutropenia following chemotherapy is a well-known risk factor [10], and some clinical predictors of pseudomonas bacteremia have been reported in patients without neutropenia [11-13]. Age over 90 years, healthcare-associated infection, indwelling central venous catheter, indwelling urinary device, and antimicrobial therapy within the previous 30 days, have been reported to be clinical predictors of pseudomonas infection.

However, no studies have focused on the clinical predictors or outcomes of pseudomonas bacteremia in patients with liver cirrhosis. The aim of this study was therefore to identify predictive factors and clinical outcomes of pseudomonas bacteremia in patients with liver cirrhosis.

## Methods

### Study design and subjects

We performed a case–control study to identify risk factors associated with pseudomonas bacteremia in cirrhotic patients. Cirrhosis was diagnosed by gastroenterologists based on patient history and physical, laboratory, and radiologic features. Cases were defined as patients with liver cirrhosis and bacteremia due to *P*. *aeruginosa* admitted to Seoul National University Hospital (SNUH) or Seoul National University Bundang Hospital (SNUBH) during the period January 2005 through December 2011. SNUH is a university-affiliated tertiary referral hospital with 1,600 beds, located in Seoul, Korea. SNUBH is also a university affiliated tertiary referral hospital and it is located in Seong-Nam, Korea. To select controls, we recruited at random cirrhotic patients with bacteremias caused by Enterobacteriaceae, who were admitted to SNUH or SNUBH in the same period. The numbers of case and control patients were the same. When patients experienced two or more episodes of bacteremia, we selected the first.

### Data collection

We reviewed electronic medical records and following data have been collected: age, sex, etiology of liver cirrhosis, liver function measured by Child-Pugh score, severity of bacteremia measured by Pitt bacteremia score, Charlson weighted index of comorbidity, associated diseases or conditions (such as diabetes, renal failure, malignancies, chronic obstructive pulmonary disease, bronchiectasis, etc.), primary focus of bacteremia (e.g. primary bacteremia, urinary tract, biliary tract, vascular catheters, and etc.), potential risk factors of pseudomonas infection (such as nosocomial or healthcare-associated infection, antibiotic exposure in recent 30 days, use of corticosteroids or anti-neoplastic agents, central venous catheter, urinary catheter, nasogastric tube, neutropenia), laboratory data at the time of diagnosis of bacteremia (such as serum creatinine, prothrombin time, bilirubin, albumin), adequacy of initial antibiotics, and 30-day mortality.

### Definitions

Community acquired bacteremia: bacteremia developed before or in the initial 48 hours of hospitalization and not due to healthcare-associated bacteremia.

Nosocomial acquisition: nosocomial bacteremia or healthcare-associated bacteremia. Nosocomial bacteremia was diagnosed when the bacteremia developed more than 48 hours after admission. We considered it healthcare-associated if any of the following conditions were present: 1) hemodialysis in the previous 30 days, 2) antibiotic treatment in the previous 30 days, 3) a history of > 48 hours hospital admission (including nursing home and long term care facility) in the previous 90 days.

Recent exposure to immunosuppressive drugs: exposure to anti-neoplastic drugs, cytotoxic agents, corticosteroids, or monoclonal antibodies used to suppress the immune system within 30 days of the onset of bacteremia.

Appropriate antibiotic treatment: at least one antibiotic shown to be effective in microbiologic tests administrated within 24 hours.

### Microbiologic test

The VITEK II automated system (bioMérieux Inc., Marcy L’Etoile, France) was used to identify bacterial organisms. In the VITEK II, system bacterial organisms are identified with standard identification cards.

### Statistics

Student’s *t*-test was used for normally distributed continuous variables, and the Wilcoxon rank sum test for non-normally distributed continuous variables. The Chi-square or Fisher’s exact test was used to analyze categorical variables. A logistic regression model was adopted to adjust for confounding variables and to identify risk factors. To this end, we performed a stepwise multivariate logistic regression analysis. All variables with *P* <0.1 in the univariate analysis were included in the multivariate analysis, and variables with *P* <0.05 were selected in the final model. SAS v9.3 (SAS Institute Inc., Cary, NC, USA) was used for all statistical analyses.

### Study approval

This study was approved by the institutional review board of the Seoul National University Hospital Medical Center (Protocol No; H-1201-002-391) according to the Helsinki Declaration. Informed consent was waived by the board.

## Results

### Study populations

During the study period, a total of 67 episodes of pseudomonas bacteremia developed in 63 patients, and 739 episodes of Enterobacteriaceae bacteremia in 593 patients. Two patients with polymicrobial bacteremia caused by *P*. *aeruginosa* and Enterobacteriaceae were excluded. Finally, 61 pseudomonas bacteremia patients were included as the case group, and 61 control patients were selected at random from the above 593 patients; in the latter, *Escherichia coli* was most common organism (29/61, 47.5%), followed by *Klebsiella pneumoniae* (22/61, 36.1%), *Enterobacter cloacae* (4/61, 6.6%), *Citrobacter freundii* (2/61, 3.3%), *Enterobacter aerogenes* (1/61, 1.6%), and *Salmonella enterica* (1/61, 1.6%).

### Predictive factors for *P*. *aeruginosa* bacteremia

The mean age (± standard deviation) of the case group was slightly lower than that of the control group: 56.8 ± 13.1 and 60.7 ± 8.9, respectively (Table 1). Males predominated in both groups. Biliary origin was more common in the case group (52.5% vs. 26.3%, *P* <0.01), as was nosocomial acquisition (83.6% vs. 54.1%, *P* < 0.01). Neither the severity of cirrhosis measured by Child-Pugh class nor the severity of comorbid conditions measured by Charlson weighted index or the severity of bacteremia measured by Pitt bacteremia score differed between the two groups. Liver transplantation was performed more frequently in the case group (32.8% vs. 16.4%, *P* = 0.04), and preexisting biliary disease was also more common (63.9% vs. 29.5%, *P* <0.01), as was the use of immunosuppressive drugs (39.3% vs. 18.0%, *P* <0.01).

**Table 1:** **Baseline and clinical characteristics of 61 cirrhotic patients with *Pseudomonas aeruginosa *bacteremia and 61 controls with Enterobacteriaceae bacteremia**

**Variable**	**Case patients**	**Control patients**	***P***
	**(n = 61)**	**(n = 61)**	
Age (mean in year ± SD)	56.8 ± 13.1	60.7 ± 8.9	0.06
Male (%)	46 (75.4)	44 (72.1)	0.68
Etiology of liver cirrhosis (%)			0.10
HBV	41 (67.2)	44 (72.1)	
HCV	6 (9.8)	11 (18.0)	
Alcohol	1 (1.6)	2 (3.3)	
Cryptogenic	11 (18.0)	4 (6.6)	
Biliary	2 (3.3)	0 (0.0)	
Source of bacteremia (%)			<0.01
Lung	4 (6.6)	1 (1.6)	
Urinary tract	1 (1.6)	6 (9.8)	
CVC	0 (0.0)	0 (0.0)	
SBP	6 (9.8)	19 (31.2)	
Biliary tree	32 (52.5)	16 (26.3)	
Abdomen^a^	7 (11.5)	3 (4.9)	
Other	2 (3.3)	1 (1.6)	
Unknown	9 (14.8)	15 (24.6)	
Onset of bacteremia (%)			<0.01
Community acquired	10 (16.4)	28 (45.9)	
Nosocomial^b^	51 (83.6)	33 (54.1)	
Child-Pugh class (%)			0.64
A	17 (27.9)	16 (26.2)	
B	24 (39.3)	22 (36.1)	
C	20 (32.8)	23 (37.7)	
Pitt bacteremia score (mean ± SD)	2.0 ± 2.6	1.4 ± 1.9	0.14
Charlson weighted index of comorbidity (mean ± SD)	5.7 ± 1.9	5.2 ± 1.7	0.16
Comorbid conditions (%)			
Liver transplantation	20 (32.8)	10 (16.4)	0.04
Biliary disease	39 (63.9)	18 (29.5)	<0.01
Diabetes mellitus	4 (6.6)	5 (8.2)	1.00
End stage renal disease	5 (8.2)	4 (6.6)	1.00
Hepatocellular carcinoma	40 (65.6)	38 (62.3)	0.71
Solid tumor other than HCC	8 (13.1)	4 (6.6)	0.36
Hematologic malignancies	3 (4.7)	0 (0.0)	0.24
Chronic obstructive pulmonary disease	1 (1.6)	0 (0.0)	1.00
Bronchiectasis	2 (3.3)	0 (0.0)	0.50
Clinical presentation (%)			
Fever	51 (83.6)	49 (80.3)	0.64
Acute renal failure	0 (0.0)	6 (9.8)	0.03
Septic shock	5 (8.2)	7 (11.5)	0.54
Co-existing conditions (%)			
Neutropenia	2 (3.3)	0 (0.0)	0.50
Immunosuppressive drug^c^	24 (39.3)	11 (18.0)	<0.01
CVC	2 (3.3)	6 (9.8)	0.27
Urinary catheter	2 (3.3)	5 (8.2)	0.44
Nasogastric tube	2 (3.3)	3 (4.9)	1.00
Previous exposure of antibiotics (%)	52 (85.3)	50 (82.03)	0.47
Polymicrobial bacteremia (%)	5 (8.2)^d^	2 (3.3)^e^	0.44
Laboratory data at the time of bacteremia			
Bilirubin (mg/dL), median (IQR)	3.7 (1.2 - 13.4)	2.2 (1.4 - 5.5)	0.34
Prothrombin time (by INR), median (IQR)	1.29 (1.11 - 1.56)	1.43 (1.21 - 1.70)	0.02
Albumin (g/dL), median (IQR)	3.1 (2.8 - 3.8)	3.0 (2.6 - 3.4)	0.30
Creatinine (mg/dL), median (IQR)	1.00 (0.81 - 1.56)	1.00 (0.90 - 1.40)	0.50
Appropriate initial antibiotic regimen (%)	18 (29.5)	40 (65.6)	<0.01
30-day mortality (%)	12 (19.7)	15 (24.6)	0.51

^a^Intra-abdominal infection excluding SBP and biliary tree infection.

^b^Includes both nosocomial bacteremia and healthcare-associated bacteremia.

^c^Includes anti-neoplastic drugs, cytotoxic drugs, and corticosteroids.

^d^Associated bacteria were *Enterococcus faecalis* (2), *Enterococcus faecium* (1), *Enterococcus gallinarum* (1), *Staphylococcus epidermis* (1).

^e^Associated bacteria were *Salmonella enterica* + *E*. *faecium* (1), *Serratia liquefaciens* + *Enterobacter cloacae* (1).

Abbreviation: *SD* standard deviation, *HBV* hepatitis B virus, *HCV* hepatitis C virus, *CVC* central venous catheter, *SBP* spontaneous bacterial peritonitis, *IQR* interquartile range, *HCC* hepatocellular carcinoma, *INR* international normalized ratio.

Age, etiology of liver cirrhosis, onset of bacteremia (community onset vs. nosocomial acquisition), liver transplantation, preexisting biliary disease, acute renal failure, recent exposure to immunosuppressive drug, and prothrombin time were selected for the multivariate logistic regression analysis. After stepwise selection, age, onset of bacteremia, preexisting biliary disease, and exposure to immunosuppressive drugs were included in the final model (Table 2). Age was negatively associated with *P*. *aeruginosa* bacteremia with an adjusted odds ratio (aOR) of 0.96 (95% confidence interval: 0.93 - 0.99, *P* = 0.03), and nosocomial acquisition was related to pseudomonas bacteremia with aOR 3.87 (95% confidence interval: 1.50 - 9.94, *P* < 0.01). Preexisting biliary disease was strongly related to pseudomonas bacteremia (aOR 4.79, 95% confidence interval: 1.92 - 10.47, *P* <0.01), as was recent exposure to immunosuppressive drugs (aOR: 3.10, 95% confidence interval: 1.23 - 7.87, *P* = 0.02).

**Table 2:** **Independent factors associated with *Pseudomonas aeruginosa *bacteremia in cirrhotic patients on the basis of a multivariate logistic regression analysis**^**a**^

**Variable**	**Adjusted odd ratios (95% CI)**	***P***
Age	0.96 (0.93 - 0.99)	0.03
Nosocomial acquisition^b^	3.87 (1.50 - 9.94)	<0.01
Biliary disease	4.79 (1.92 - 10.47)	<0.01
Immunosuppressive drug	3.10 (1.23 - 7.82)	0.02

^a^Variables with *P* <0.1 in the univariate analysis were included in the multivariate logistic regression analysis.

^b^Includes both nosocomial bacteremia and healthcare-associated bacteremia.

Abbreviation: *CI* confidence interval.

### Clinical outcomes and the impact of inadequate antibiotics

In the case group the frequency of appropriate initial antibiotic regimens was considerably lower than in the control group: 29.5% vs. 65.6% (*P* <0.01). However, 30-day mortality did not differ: 12/61 (19.7%) in the case group, 15/61 (24.6%) in the control group (*P* = 0.51). In the case group, 18/61 (29.5%) of the patients received adequate initial antibiotic therapy. Of these, eleven received beta-lactam antibiotics, and seven, quinolones. The clinical characteristics and outcomes in the case group according to adequacy of empirical antibiotics are shown in Table 3. 30-day mortality was lower in the patients who were prescribed adequate initial antibiotic agent(s) than in those who were not, (11.1% vs. 23.3%), but the difference was not significant (*P* = 0.48).

**Table 3:** **Outcomes and impact of inadequate antibiotics among 61 cirrhotic patients with *Pseudomonas aeruginosa *bacteremia**

**Variable**	**Initial therapy**		***P***
	**Adequate (n = 18)**	**Inadequate (n = 43)**	
Age (mean in year, SD)	49.1 ± 15.5	60.0 ± 10.5	0.01
Male (%)	13 (72.2)	33 (76.7)	0.75
Child-Pugh score (mean ± SD)	7.1 ± 2.1	8.7 ± 2.4	0.02
Pitt bacteremia score (mean ± SD)	1.3 ± 1.9	2.3 ± 2.9	0.18
30-day mortality (%)	2 (11.1)	10 (23.3)	0.48

Abbreviation: *SD* standard deviation, *IQR* interquartile range.

## Discussion

In this study, nosocomial acquisition was an important predictor of pseudomonas bacteremia with aOR 3.87 (95% confidence interval: 1.50 - 9.94, *P* <0.01). *P*. *aeruginosa* is a significant cause of infection in patients exposed to hospital environments; the second most common cause of nosocomial pneumonia, and the third most common cause of nosocomial urinary tract infection [14]. Thus this finding is plausible, and consistent with previous reports [11,13,15,16].

It is notable that pseudomonas bacteremia was significantly associated with the presence of biliary disease in cirrhotic patients, with aOR 4.79 (95% confidence interval: 1.92 - 10.47, *P* <0.01). *P*. *aeruginosa* is one of the most frequent etiologic organisms of bacteremic biliary tract infection [17-19]. In our study, 33 of the 39 case patients with biliary tract disease had preexisting biliary catheters, including percutaneous transhepatic biliary drains, endoscopic retrograde biliary drains, and endoscopic nasobiliary drains. *P*. *aeruginosa* can form firm biofilms on foreign material and cause foreign material-associated infections [14,20,21]. Thus biliary catheters may play a role in the origin of biliary sepsis involving *P*. *aeruginosa* in patients with biliary diseases, and the results of previous studies support this notion [22-24]. Twenty of the 61 (32.8%) patients with pseudomonas bacteremia had received liver transplants. During such operations, biliary-to-biliary anastomosis is made, and biliary catheters are frequently used. This suggests that clinicians should consider prescribing empirical antibiotics with anti-pseudomonas activity when gram negative bacteremia develops in cirrhotic patients with biliary diseases or preexisting biliary catheters.

It is well known that immune deficiency conditions, such as HIV infection, neutropenia, and use of immunosuppressive agents, are risk factors for pseudomonas infection [14]. Recent use of immunosuppressive agents was also associated with pseudomonas bacteremia in this study. We found that the mean age of patients with pseudomonas bacteremia was lower than that of patients with Enterobacteriaceae bacteremia. However the difference was too small to have clinical significance (aOR: 0.96, 95% confidence interval: 0.93 - 0.99, *P* = 0.03).

Inappropriate antibiotic therapy is known to result in poor prognoses in *P*. *aeruginosa* bacteremia [6-9,12,25,26]. In our study, inappropriate initial antibiotics were prescribed more frequently in the case group, but mortality was similar in the two groups. We think that the difference between our findings and previous ones may be due to differences in the study populations: in the earlier studies, pneumonia, non-hepatobiliary tract origin, presence of neutropenia, and initial presentation with septic shock, were prognostic factors for poor outcomes in patients with pseudomonas bacteremia [7,11]. In our study, the most common source of bacteremia was the biliary tract, and only a small proportion of the patients had neutropenia. Our mortality findings are similar to those of Joo et al., who found similar mortality in pseudomonas bacteremia and Enterobacteriaceae bacteremia [11]. In that study the pseudomonas bacteremia originated from biliary tract infections in 50% of the patients. It is well known that interventions to relieve biliary tree obstruction are the mainstay of treatments for biliary tree infection [14].

As far as we know, this is the first study examining the clinical predictors of pseudomonas bacteremia in cirrhotic patients. It has several limitations. First, it is a retrospective study, and there may be unidentified confounding factors. Second, although we enrolled patients in two tertiary hospitals over seven years, the study population was relatively small, and we could not assess the significance of small differences. Third, control group was more heterogeneous than the case group, because the control group included patients infected with a variety of species of the family Enterobacteriaceae.

## Conclusions

Nosocomial acquisition, biliary disease, and exposure to immunosuppressive agents are significantly related to *P*. *aeruginosa* bacteremia in cirrhotic patients, compared to cirrhotic patients with bacteremia caused by Entrobacteriaceae.

## Competing interests

All authors declare that they have no competing interests.

## Authors’ contributions

KNJ designed and supervised the study. BJH contributed to the design of the study. BJH and JY drafted the manuscript. JY, CS, KCJ, SKH, KHB, and LH participated in collecting data. CPG, KES, and PSW made contributions to the interpretation of data. PWB, LH, and OM revised the manuscript critically for important intellectual content. All authors read and approved the final manuscript.

## Pre-publication history

The pre-publication history for this paper can be accessed here:

http://www.biomedcentral.com/1471-2334/13/332/prepub
